# Association between sleep quality with maternal and neonatal outcomes during the covid-19 pandemic

**DOI:** 10.1186/s12884-024-06479-y

**Published:** 2024-04-19

**Authors:** Maryam Akbari, Sara EsmaeilzadehSaeieh, Malihe Farid, Arman Shafiee, Mahmood Bakhtiyari, Touran Bahrami Babaheidari, Mansoureh Yazdkhasti

**Affiliations:** 1 Department of Midwifery, School of Medicine, Alborz University of Medical Sciences, Karaj, Iran; 2 Department of Midwifery, School of Medicine, Social Determinants of Health Research Center, Alborz University of Medical Sciences, Hasan Abad Blvd ooge St, P.O. Box: 3149779453, Karaj, Iran; 3 Department of Community Medicine, School of Medicine, Alborz University of Medical Sciences, Karaj, Iran

**Keywords:** Sleep disorder, COVID-19 pandemic, Pregnant women, Maternal outcomes, Neonatal outcomes

## Abstract

**Aim:**

Sleep disorders during pregnancy can impact maternal and neonatal outcomes. The objective of this study is to examine the relationship between sleep quality and maternal and neonatal outcomes during the COVID-19 pandemic.

**Method:**

This prospective cohort study was conducted at the Educational-Therapeutic Center of Shohadaye Yaftabad Referral Hospital in Tehran, Iran, from December 2020 to September 2022. A total of 198 eligible participants were randomly assigned to either the sleep disorders group or the no sleep disorders group. Data were collected through demographic questionnaires, the Corona Disease Anxiety Scale (CDAS) questionnaire, the Pittsburgh Sleep Quality Index (PSQI), and the checklist for maternal and neonatal outcomes.

**Results:**

At baseline, the sleep disorders and no sleep disorders groups were similar in terms of age, body mass index (before pregnancy), education level, employment status, gravida, parity, abortion, and history of COVID-19. Within the sleep disorders group, there was a statistically significant, direct linear correlation between sleep disorders and FBS 34–36 weeks (*r* = 0.33, *P* < 0.001) as well as Corona Disease Anxiety (CDA) (*r* = 0.35, *P* < 0.001). The linear regression results indicated that for every unit increase in sleep disorders, the risk of FBS 34–36 weeks increased by 1.09 times (β = 1.09, *P* < 0.001). Additionally, sleep disorders increased the risk of CDA by 1.36 times (β = 1.36, *P* < 0.001). The results showed no statistically significant differences in terms of birth weight, type of delivery (vaginal or cesarean section), gestational age (preterm or full term), length of labor stages (first and second stage), Apgar score at minutes 1 and 5, and NICU admission between the two groups.

**Conclusion:**

Based on the results, a certain degree of correlation exists between sleep quality and FBS at 34–36 weeks and CDA. These findings underscore the need for future public health guidelines to formulate detailed strategies to improve sleep quality during the COVID-19 pandemic.

## Introduction

Sleep is closely tied to various aspects of human physical and mental health [1]. The main purposes of sleep are to conserve energy in brain cells, establish balance in brain synapses [2], and protect the body against inflammation [3]. As individuals age, their sleep patterns change, resulting in decreased duration and consistency, as well as an increased prevalence of sleep disorders in adulthood [4]. Women are more susceptible to sleep disorders than men [5]. Throughout a woman’s life, biological cycles such as menstruation, pregnancy, and menopause can lead to changes in the structure and quality of sleep [6]. Pregnancy, in particular, can cause or worsen sleep disorders due to anatomical, physiological, and hormonal changes [7]. These changes typically begin in the first trimester, and pregnant women become more susceptible to sleep disorders as the pregnancy progresses [8]. The estimated prevalence of sleep disorders during pregnancy is 45.7% [9], with a reported prevalence of 20.7% in Iran [10].

Sleep disturbances can hurt other bodily systems, including the endocrine, metabolic, and nervous systems [11, 12], by increasing activity in the sympathetic system and markers of inflammation and oxidative stress [7, 13]. Research has shown a link between sleep disorders, elevated blood sugar levels, and the risk of diabetes in the general population [14, 15]. Since pregnancy itself is associated with insulin resistance [16], changes in sleep patterns may further increase pregnant women’s likelihood of experiencing high blood sugar levels [17, 18]. Pregnancy is accompanied by numerous immunological changes, leading to a chronic state of immunosuppression to protect the fetus [19]. Sleep disorders can upregulate inflammatory markers [20], increasing the risk of premature birth and preeclampsia [21]. Additionally, episodes of hypoxia and apnea resulting from breathing disturbances during sleep can lead to damage to the endothelium and reduced oxygen supply to the fetus, contributing to preeclampsia and negative neonatal outcomes like intrauterine growth restriction (IUGR) [22]. Multiple studies have demonstrated that sleep disorders can impact maternal outcomes, including hypertension, preeclampsia, miscarriage, premature birth, and cesarean delivery, as well as neonatal outcomes such as low birth weight, stillbirth, and Apgar score. Furthermore, experiencing sleep disturbances during pregnancy may increase the likelihood of postpartum depression. A study found that during the COVID-19 pandemic, the prevalence of anxiety and depression in pregnant women was 15.8% and 25.8%, respectively [23–25].

The connection between sleep and physical and mental health has been well-documented [26]. Anxiety during pregnancy [27] is one of the stressors that can impact a mother’s mental well-being. Natural events or sensitive circumstances can contribute to maternal anxiety, and the COVID-19 pandemic is one such major event [28]. In December 2019, a genetically modified virus from the coronavirus family known as SARS-CoV-2 emerged, causing a global pandemic known as Covid-19 [29]. The first study investigating the relationship between sleep and the COVID-19 pandemic was conducted in China, revealing an 18.2% prevalence of sleep disturbances in the general population [30]. The term “coronasomnia” has been coined to describe sleep problems associated with the stress caused by the COVID-19 pandemic [31]. Studies have shown an increase in the incidence of sleep disturbances among pregnant women during the Covid-19 pandemic [32, 33]. Given the high prevalence of this disease and the escalating morbidity and mortality rates worldwide, there has been a rise in the prevalence of psychological disorders such as anxiety, fear, depression, emotional changes, and insomnia among healthcare workers, pregnant women, and the general population. It is reasonable to expect that pregnant women may experience heightened sleep disturbances due to the social distancing and isolation measures implemented during the COVID-19 pandemic [29, 34, 35]. The present study aims to examine the association between sleep quality and maternal and neonatal outcomes during the COVID-19 pandemic.

## Method

This prospective cohort study was conducted at Shohadaye Yafetabad Medical Referral Hospital in Tehran, Iran, from December 2020 to September 2022. The study included 198 eligible candidates who received the code of ethics (ABZUMS.REC.1400.077) and provided written consent. In this study, we followed pregnant women eligible from 24 weeks to 40 weeks until delivery and 6 h after delivery.

The inclusion criteria were as follows: pregnancy during the COVID-19 pandemic, gestational age between 24 and 40 weeks, singleton pregnancy, and low-risk pregnancy based on screening in the first and second trimesters. The exclusion criteria included high blood pressure during pregnancy, a history of overt diabetes or gestational diabetes, smoking, use of narcotics and psychoactive substances, as well as a history of anxiety, depression, or other mental illnesses.

### Sampling and sample size

Simple randomization was performed using the free software package Random Number Generator (https://www.random.org). The participants were divided into two groups, each containing 99 participants.

A sample size of 198 achieves 100% power to detect a change in slope from 0 under the null hypothesis to 1.09 under the alternative hypothesis when the statistical hypothesis is two-sided, the significance level is 0.05, the standard deviation of PSQI is 5, the standard deviation of FBS levels is 10 mg/dl, the standard deviation of residuals is 8.38436, and R² is 0.29703.

### Data Collection

Data were collected using three questionnaires: Demographics, Pittsburgh Sleep Quality Index, and Corona Disease Anxiety Scale. The Demographics questionnaire included questions on age, body mass index (BMI), education level, employment status, gravida, parity, abortion, and history of COVID-19.

### Pittsburgh Sleep Quality Index (PSQI)

This questionnaire (Persian version of PSQI) consists of 7 subscales: subjective sleep quality, sleep latency, sleep duration, habitual sleep efficiency, sleep disturbances, use of sleeping medication, and daytime dysfunction. A score higher than 5 indicates a sleep disorder. In our study, mothers were categorized as having sleep disorders (> 5) or no sleep disorders (≤ 5).

The reliability of the questionnaire was reported as 0.83 using Cronbach’s alpha [36]. The sensitivity and specificity of the instrument in Iran have been reported to be 0.87 [37].

### Corona Disease anxiety scale (CDAS)

High scores on the CDAS indicate a higher level of anxiety. The reliability was evaluated at 0.87 for mental symptoms and 0.91 for physical symptoms using Cronbach’s alpha test. It should be noted that the CDAS was primarily developed for the Iranian population [38].

### Checklist

The checklist for maternal and newborn outcomes includes FBS at 24–28 weeks, FBS at 34–36 weeks, length of the first phase of labor (from the beginning of dilation 4–6 cm to complete dilation), length of the second phase of labor (from full dilation until delivery), birth weight, first and fifth-minute Apgar scores, gestational age (preterm or full term), type of delivery (natural or cesarean section), and NICU admission.

### Statistical analysis

Statistical analysis was conducted using SPSS 23 software. Data normality was assessed using Skewness and Kurtosis. For variables with a normal distribution, independent parametric t-tests and Pearson tests were used. For variables without a normal distribution, Spearman’s correlation was used. Chi-square and Fisher’s exact tests were used for qualitative variables.

Before conducting the linear regression test, the correlation between variables was analyzed using Spearman and Pearson tests. Pearson correlation (r) measures linear dependence between two variables, while Spearman’s correlation (rho) measures the strength and direction of monotonic association. In the linear regression test (Enter Method), PSQI was considered the independent variable, and maternal and newborn outcomes were the dependent variables. A p-value < 0.05 was considered statistically significant.

## Results

The demographic information of 198 participants was analyzed (*n* = 99 individuals in each group). The two groups showed similarities in terms of age, body mass index (before pregnancy), education level, employment status, gravida, parity, abortion, and history of COVID-19 (Table 1).

**Table 1:** Baseline sociodemographic and gestational characteristics in two groups

Variable		No sleep disorders Group *n*=99	Sleep disorders Group *n*=99	P-value
Age (Y)		27.76±5.671	28.44±6.105	0.07^a^
BMI (kg/m^2^)		27.81±4.10	28.66±4.300	0.16 ^a^
Education level	Primary	2 (2%)	5 (5.1%)	0.676 ^b^
	Diploma	30 (30.3%)	30 (30.3%)	
	Bachelor	48 (48.5%)	47 (47.5%)	
	Masters	19 (19.2%)	17 (17.1%)	
employment status	Unemployed	93 (93.9%)	95 (96%)	0.516 ^b^
	Employed	6 ( 6.1%)	4 ( 4%)	
Gravida	1-3	89 (89.9%)	87 (87.9%)	0.651 ^b^
	4-6	10 (10.1%)	12 (12.1%)	
Parity	0-2	94 (94.9%)	96 (97.0%)	0.498^c^
	3-4	5 (5.1%)	3 (3.0%)	
Abortion	0-1	95 (96%)	96 (97.0%)	1.000^c^
	2-3	4 (4%)	3 (3.0%)	
History of Covid-19	Yes	39 (39.4%)	49 (49.5%)	0.198^c^
	No	60 (60.6%)	50 (50.5%)	
Gestational week	24-28	30(30%)	26 (26.3%)	0.772^b^
	29-32	3(3.1%)	7(7.7%)	
	33-36	28(28%)	28(28.4%)	
	37-40	38(38%)	38(37.6%)	

^a^_Independent t−test_

^b^_Chi−square_

^c^_Fisher exact test_

_Y; year, BMI; Body Mass Index_

In the sleep disorder group, the results of the Pearson Correlation test revealed a statistically significant and direct linear correlation between sleep disorders and FBS at 34–36 weeks (*r* = 0.33, *P* < 0.001). Additionally, the Spearman test results demonstrated a statistically significant and direct linear correlation between sleep disorders and CDA (*r* = 0.35, *P* < 0.001). However, there was no statistically significant correlation between sleep disorders and FBS at 24–28 weeks, birth weight, length of the First Phase of Labor (LFPL), length of the Second Phase of Labor (LSPL), Apgar score at 1 min, and Apgar score at 5 min. In the group without sleep disorders, no statistically significant correlation between sleep disorders and maternal and neonatal outcomes was observed (Table 2).

**Table 2:** Correlation of sleep disorders with maternal and newborn outcomes in two groups

Variable	No sleep disorders group *n* = 99 Mean ± SD	PSQI Correlation coefficient	P-value	Sleep disorders group *n* = 99 Mean ± SD	PSQI Correlation coefficient	P-value
FBS 24–28 week (mg/dl)	80.21 ± 7.39	-0.04 ^b^	0.67	93.68 ± 7.71	0.26 ^a^	0. 47
FBS 34–36 week (mg/dl)	79.66 ± 8.85	0.03 ^a^	0.42	95.86 ± 10.11	0.33 ^a^	0.001 *
CDA	7.39 ± 5.73	0.01 ^a^	0.27	11.91 ± 9.20	0.35 ^b^	0.001*
LFPL (min)	156.25 ± 64.31	0.47 ^a^	0.78	173.21 ± 78.50	0.016 ^a^	0.89
LSPL (min)	22.92 ± 11.91	0.14 ^b^	0.41	25.00 ± 13.15	0.15 ^a^	0.35
Birth Weight (g)	3318.33 ± 47.96	0.30 ^a^	0.76	3270.11 ± 41.73	0.13 ^a^	0.18
Apgar 1st min	8.77 ± 0.47	-0.18 ^b^	0.06	8.86 ± 0.35	0.033 ^b^	0. 74
Apgar 5th min	9.95 ± 0.26	-0.05 ^b^	0.96	9.98 ± 0.14	0.082 ^b^	0.39

^a^ Pearson correlation (r)

^b^ *Spearman’s correlation(rho)*

*P < 0.05

PSQI; Pittsburgh Sleep Quality Index, FBS; Fast Blood SugarCDA; Corona Disease Anxiety, LFPL; length of the First Phase of Labor, LSPL; length of the Second Phase of Labor, g; gram, min; minute

Variables exhibiting a statistically significant PSQI correlation underwent linear regression analysis. The results of the linear regression demonstrated that for every unit increase in sleep disorders, the risk of FBS at 34–36 weeks increased by 1.09 times (β = 1.09, *P* < 0.001). Moreover, sleep disorders increased the risk of CDA by 1.36 times (β = 1.36, *P* < 0.001) (Table 3).

**Table 3:** Linear regression explaining sleep disorders by variables

Independent Variable	β	t- value	P- value
PSQI^c^	1.09^a^	3.57 ^a^	0.001* ^a^
	1.36^b^	4.21^b^	0.001*^b^

Dependent Variable; FBS 34–36 weeks (mg/dl), ^b^ Dependent Variable; CDA (Corona Disease Anxiety),

^c^ Independent variable; PSQI (Pittsburgh Sleep Quality Index)

β; Beta) The regression coefficient(

*P < 0.05

The Chi-square test results showed no statistically significant difference between the two groups regarding gestational age (pre-term and full-term), type of delivery, and NICU admission (Table 4).

**Table 4:** Comparison of neonatal-gestational outputs in two groups

Variable		Sleep disorders Group *n* = 99		No sleep disorders Group *n* = 99		p-value ^a^
		Frequency (N)	Percent (%)	Frequency (N)	Percent (%)	
GA	Preterm	6	6.1	5	5.1	0.756
	Full term	93	93.9	94	94.9	
TD	NVD	39	52.7	35	47.3	0.557
	CS	60	51.6	64	48.4	
NICU admission	Yes	6	8.1	8	6.1	0.579
	No	93	91.9	91	93.9	

^a^ Chi-Square Tests

GA; Gestational Age, TD; Type of Delivery, NVD; Natural Vaginal Delivery, CS: Cesarian Section, NICU; Neonatal intensive care unit, N: Number

## Discussion

This prospective cohort study aimed to assess the effect of sleep disorders on maternal and neonatal outcomes during the COVID-19 pandemic involving 198 eligible volunteers. Based on the findings, there was some degree of correlation between sleep disorders and FBS at 34–36 weeks and CDA. Our study reported that for every unit increase in sleep disorders, the risk of FBS at 34–36 weeks and CDA increased by 1.09 and 1.36 times, respectively.

Sleep disorders induce changes in the hormonal cycle and neurotransmitters, causing endocrine changes by increasing sympathetic activity and disrupting circadian rhythms. Additionally, they contribute to endothelial damage and placental dysfunction by increasing inflammatory response and causing interruptions in oxygen supply. Therefore, sleep disorders can impact maternal and newborn outcomes [23, 24, 39, 40].

Meanwhile, minor changes in sleep quality expose mothers to more insulin resistance [17, 18]. Various theories support the link between insufficient sleep and sleep disorders with increased insulin resistance. Obstructive Sleep Apnea (OSA) increases oxidative stress and downregulates antioxidants. Studies have shown increased levels of oxidative stress in women with gestational diabetes compared to non-diabetic women. Prolonged sleep disorders are associated with chronically elevated cortisol levels, suppressing insulin secretion [41]. Changes in the hormonal cycle, oxidative stress, inflammatory factors (interleukin-6 and tumor necrosis factor alpha), and regulation signals for adipogenesis can cause disturbances in glucose metabolism [7, 11–13].

The increased prevalence of sleep disorders during the third trimester of pregnancy [42] is associated with increased FBS. As the pregnancy progresses and the uterus grows in the third trimester, the pressure on the lungs and bladder increases, leading to frequent urination. Therefore, pregnant mothers sometimes have to wake up several times. Additionally, increased estrogen during pregnancy decreases the diameter of the bronchi and respiratory tracts, increasing the likelihood of respiratory disturbances. These factors have been reported to increase the risk of glucose metabolism disorders and sleep disorders [43]. In a study where gestational diabetes was considered the main outcome of sleep disorders, gestational diabetes was found to be a suitable predictor for sleep disorders in pregnant mothers. In other words, the prevalence of sleep disorders increases with an increase in FBS levels [18]. The relationship between sleep disorders and gestational diabetes has been reported in other studies. Even partial sleep deprivation over one night increases insulin resistance, which can, in turn, increase blood sugar levels. As a result, a lack of sleep has been associated with diabetes, a blood sugar disorder [10, 39, 44]. However, other studies have not confirmed this relationship [43, 45]. The relationship between sleep disorders and diabetes has been observed in the first trimester of pregnancy but not in the middle of pregnancy [39, 46].

According to objective evaluations, structural changes following sleep disturbances in the third trimester of pregnancy, especially in the last month (due to the greater possibility of abdominal pain or false labor, fatigue, childbirth anxiety, and muscle spasms), have been observed at a higher rate compared to non-pregnant women. These structural changes include an increase in sleep onset latency, an increase in night awakenings, a decrease in sleep duration, a decrease in habitual sleep efficiency due to night awakenings, an increase in the first stage of sleep, and a decrease in the rapid eye movement (REM) stage of sleep in pregnant women [47]. Decreased sleep quality, insufficient night sleep, daytime sleepiness, insomnia symptoms, OSA, and restless leg syndrome are among the sleep disorders experienced by pregnant women [48]. Decreased sleep quality is more frequently seen in mothers over 30 years old, in the third trimester of pregnancy, in nulliparous mothers, in mothers experiencing depression, in mothers playing the role of the mother alone, in mothers lacking support from the family, in mothers with negative mental self-image, in mothers with a high level of stress, in mothers with gestational hypertension, and in mothers exposed to environmental factors such as the noise of other children, pets, or inappropriate bedroom conditions [49]. Maternal obesity is also associated with increased sleep disorders. The prevalence of OSA increases with increasing body mass index. One study reported that the prevalence of OSA is 15–20% in obese pregnant women [9].

A decrease in sleep quality, a decrease in sleep health, and an increase in sleep disorders affect mental-social health [50]. Anxiety and stress affect many physiological aspects of the body, including the sleep-wake cycle, by disrupting the hypothalamus-pituitary-adrenal axis [51]. When we experience anxiety, sleep quality is reduced, and this reduction increases with increasing anxiety and sleep deprivation [52]. Sleep disorders, as a stressful factor, can increase stress and anxiety during pregnancy. Natural conditions and events such as the Covid-19 pandemic have made pregnant women more anxious [28, 53]. It has been reported that the increase in sleep disorders during pregnancy is associated with increased anxiety during the Covid-19 period. The fear of the unknowns of the Covid-19 pandemic and the lack of sufficient knowledge about its impact on pregnancy and neonates is a vital stressor that has affected sleep quality during pregnancy and has been associated with increased sleep disorders. On the other hand, sleep disorders also lead to a significant increase in anxiety levels. Therefore, sleep disorders and anxiety during pregnancy mutually affect each other [32, 33]. Coronasomnia has been reported to affect 18.2–57.1% of the general population [30, 54]. An increase in anxiety can be associated with a further increase in coronasomnia. Normal pregnancy is associated with an increase in the level of the noradrenaline hormone at night. Coronasomnia during pregnancy is associated with a more significant increase in noradrenaline. This is considered a factor that increases the risk of gestational diabetes, anxiety, gestational hypertension, preeclampsia, and premature birth. Pregnancy is associated with many immunological changes, and a chronic immunosuppressive state is created to preserve the fetus. Sleep disorders lead to dysregulations in several immune functions. It takes several days to restore normal immune system function even after short sleep deprivation. Sleep deprivation similarly leads to increased levels of stress and cortisol [19]. High levels of phobic anxiety are associated with increased levels of leptin and inflammatory markers. The high plasma leptin levels found in gestational diabetes may be potentiated by leptin resistance at a central level, and obesity-associated inflammation plays a role in this leptin resistance [20, 21].

One study found no relationship between sleep disorders and an increased risk of premature birth [41]. However, another study found a relationship between sleep disorders and premature birth and cesarean Sect. [46], low birth weight [45], first-minute Apgar score [46], and SGA [47]. Sleep duration is another factor that affects maternal and newborn outcomes. Reducing sleep hours is also considered a sleep disorder. As observed, reducing sleep hours to less than 7 h has been associated with increased premature birth and SGA in the third trimester [46, 55]. Sleep onset latency in early pregnancy and the decrease in sleep duration in the second trimester, along with other factors that cause sleep disorders in pregnant mothers, are more likely to be associated with lower birth weight [56, 57]. Our study found no correlation between sleep disorders and FBS at 24–28 weeks, length of the first and second stage of labor, birth weight, and first and five-minute Apgar scores. Additionally, there was no difference between the two groups regarding gestational age (pre-term and full-term), type of delivery, and NICU admission.

### Limitations

One limitation of this research was the reduced number of in-person prenatal care visits during the Covid-19 pandemic. Attempts were made to facilitate the sampling process through prior coordination with the participants.

## Conclusion

Based on the results, there is some degree of correlation between sleep quality and FBS at 34–36 weeks and Corona Disease Anxiety (CDA). These findings emphasize the need for future public health guidelines to formulate detailed strategies to enhance sleep quality during the Covid-19 pandemic. Therefore, it is suggested that appropriate interventions be implemented to improve sleep health in pregnant mothers under stressful situations such as the Covid-19 pandemic, aiming to reduce anxiety in pregnant mothers and manage blood sugar levels in the third trimester of pregnancy.

## Data Availability

The datasets generated and analyzed during the current study are not publicly available due to limitations of ethical approval involving patient data and anonymity. Still, they are available from the corresponding author at a reasonable request.
